# Laminin and Type IV Collagen Isoform Substitutions Occur in Temporally and Spatially Distinct Patterns in Developing Kidney Glomerular Basement Membranes

**DOI:** 10.1369/0022155413501677

**Published:** 2013-10

**Authors:** Dale R. Abrahamson, Patricia L. St. John, Larysa Stroganova, Adrian Zelenchuk, Brooke M. Steenhard

**Affiliations:** Department of Anatomy and Cell Biology and the Kidney Institute, University of Kansas Medical Center, Kansas City, Kansas

**Keywords:** glomerular filtration barrier, glomerular endothelial cells, podocytes

## Abstract

Kidney glomerular basement membranes (GBMs) undergo laminin and type IV collagen isoform substitutions during glomerular development, which are believed to be required for maturation of the filtration barrier. Specifically, GBMs of earliest glomeruli contain laminin α1β1γ1 and collagen α1α2α1(IV), whereas mature glomeruli contain laminin α5β2γ1 and collagen α3α4α5(IV). Here, we used confocal microscopy to simultaneously evaluate expression of different laminin and collagen IV isoforms in newborn mouse GBMs. Our results show loss of laminin α1 from GBMs in early capillary loop stages and continuous linear deposition of laminin bearing the α5 chain thereafter. In contrast, collagen α1α2α1(IV) persisted in linear patterns into late capillary loop stages, when collagen α3α4α5(IV) first appeared in discontinuous, non-linear patterns. This patchy pattern for collagen α3α4α5(IV) continued into maturing glomeruli where there were lengths of linear, laminin α5-positive GBM entirely lacking either isoform of collagen IV. Relative abundance of laminin and collagen IV mRNAs in newborn and 5-week-old mouse kidneys also differed, with those encoding laminin α1, α5, β1, β2, and γ1, and collagen α1(IV) and α2(IV) chains all significantly declining at 5 weeks, but α3(IV) and α4(IV) were significantly upregulated. We conclude that different biosynthetic mechanisms control laminin and type IV collagen expression in developing glomeruli.

## Introduction

The kidney glomerulus is a capillary tuft at the anterior end of each nephron. Every day in humans, ~180 liters of plasma fluid pass from the blood across the glomerular capillary walls and into the proximal convoluted tubules of nephrons, where most of the filtrate volume is then reabsorbed. Ordinarily, plasma albumin (~68 kDa) and larger proteins, such as the immunoglobulins, do not appreciably penetrate the glomerular capillary wall and enter the filtrate, and these proteins are retained in the circulation instead. Smaller plasma proteins, as well as trace amounts of albumin, cross the glomerular filtration barrier, but these will also usually be reabsorbed by the tubular epithelium, so that essentially protein-free urine will be excreted. However, diseases affecting the glomerular capillary wall often lead to the loss of large amounts of plasma proteins into the urine, a condition called proteinuria. If this occurs chronically, it usually results in end stage renal failure, requiring dialysis and/or transplantation. The glomerular filtration barrier comprises three interrelated structural elements: the glomerular endothelium with its associated glycocalyx/surface coat; the visceral epithelial cells of Bowman’s capsule, which are also called podocytes; and the glomerular basement membrane (GBM), which is a mat of extracellular matrix lying between the glomerular endothelial cell and podocyte layers (Haroldsson et al. 2008).

Throughout the body, basement membranes are adherent to the basal layers of epithelial cells, including the vascular endothelium, and surround all Schwann cells of peripheral nerves, all muscle cells, and adipocytes, separating these cells from the adjacent stroma. The GBM is unusual in that it is lined by two distinctly different epithelial cell types on opposite surfaces. Fenestrated glomerular endothelial cells line the inner GBM surface and podocytes line the outer GBM surface. Like all basement membranes, the GBM is composed of polymerized networks of laminin and type IV collagen and also contains nidogen and heparan sulfate proteoglycans (Abrahamson 2012; Miner 2012). Unlike most basement membranes, however, the composition of the GBM changes during glomerular development. Specifically, GBMs of the earliest, most immature nephrons contain laminin α1β1γ1 (LM-111) heterotrimers, whereas those of maturing glomeruli contain laminin α5β2γ1 (LM-521), and this is the only laminin isoform found in GBMs of adults (Miner 2011; Abrahamson 2012). Laminin α5β1γ1 (LM-511) has also been shown to occur transiently in immature GBMs (Miner et al. 1997; Miner 2011). Similarly, the most immature GBMs of early nephrons contain networks of collagen α1α2α1(IV), and those of fully mature nephrons contain collagen α3α4α5(IV) (Miner 1998). Post-fixation immunoelectron microscopy has shown that the laminin α1, α5, β1, and β2 chains are all derived from developing glomerular endothelial cells as well as podocytes (St. John and Abrahamson 2001). On the other hand, immunoelectron microscopy and metanephric grafting experiments show that endothelial cells and podocytes both secrete collagen α1α2α1(IV), but collagen α3α4α5(IV) originates solely from podocytes (Abrahamson et al. 2009).

Reasons that the GBM undergoes laminin and type IV collagen isoform substitutions are not fully understood. However, genetic diseases affecting humans, and those induced in mice, suggest that the GBM isoform transitions are necessary for attainment and maintenance of the highly differentiated state exhibited by glomerular endothelial cells and podocytes and for establishment of the glomerular filtration barrier (Abrahamson 2012). For example, mice with deletion of the Lama5 gene, which encodes laminin α5, die before birth with neural tube and placental vascular defects, among other problems (Miner et al. 1998), and glomeruli fail to develop in embryonic kidneys of these mutants (Miner and Li 2000). Mutations to the human LAMA5 gene have not yet been described, probably because they, too, result in embryonic lethality. When Lamb2 (encoding the laminin β2 chain) is knocked out in mice, mutants become massively proteinuric a few weeks after birth, and there is loss of podocyte foot processes and abnormalities in neuromuscular junctions, which is another site where laminin β2 is normally found (Noakes et al. 1995). Mutations to the human LAMB2 gene have been shown to cause Pierson syndrome, resulting in a familial nephrosis, eye abnormalities, and neonatal respiratory failure (Zenker et al. 2004). Alport syndrome is one of the most extensively studied diseases of the GBM in humans and is caused by mutations to one or more of the COL4A3, COL4A4, or COL4A5 genes, which encode the collagen α3(IV), α4(IV), and α5(IV) chains, respectively (Hudson et al. 2003; Gubler 2008). As a result of these mutations, the collagen α3α4α5(IV) network fails to form, and there is retention of collagen α1α2α1(IV) in the GBM. This typically results in focal thickening, thinning, and splitting of the GBM, loss of podocyte foot processes, progressive proteinuria, and in many affected individuals, renal failure. Mice with a genetic deletion of the COL4A3 gene have proven to be a reliable experimental model of human Alport disease (Cosgrove et al. 1996; Miner and Sanes 1996).

Because the accurate replacement of laminin and type IV collagen isoforms is so crucial for glomerular structure and function, we investigated the substitution of these GBM proteins in detail. For the experiments described here, we directly evaluated the developmental timing and spatial patterns for laminin and collagen IV isoform substitutions in developing mouse GBMs using dual label scanning confocal microscopy. Our results show that, during GBM assembly, the laminin isoform transition preceded that for type IV collagen and that there were segments of laminin-positive basement membrane entirely lacking collagen IV.

## Materials & Methods

### Immunofluorescence Microscopy

All experiments involving animals were carried out by protocols approved by the University of Kansas Medical Center Institutional Animal Care and Use Committee (Animal Care and Use Committee approval no. 2011-1972) and as stipulated by the National Institutes of Health. Newborn (1–3 days old) and 5- and 8-week-old 129/SvJ mice were killed using deep anesthesia with halothane or isoflurane followed by cervical dislocation and decapitation. Kidneys were removed, immersed in Tissue-Tec O.C.T. Compound (Electron Microscopy Sciences; Hatfield, PA), and frozen in isopentane chilled in a dry ice-acetone bath. Sections, 8-µm thick, were cut at -20C in a cryostat, picked up on Superfrost Plus microscope slides (Fisher Scientific; Pittsburgh, PA), and air-dried. Sections were fixed with freshly prepared 0.2% paraformaldehyde in phosphate-buffered saline (PBS) for 10 min at room temperature, washed three times with PBS, and then permeabilized by incubation with 0.5% Triton X-100 in PBS for 5 min at room temperature. Slides were washed three times in PBS and double labeled with mixtures of anti-laminin and/or anti-collagen IV antibodies (Table 1) for 15 min at room temperature. After washing three times with PBS, slides were then incubated with appropriate secondary antibody mixtures (Table 1) for 15 min and washed three times with PBS. Slides incubated with secondary antibody mixtures alone served as controls. Slides were then coverslipped with ProLong Gold Antifade Reagent plus DAPI (Life Technologies/Molecular Probes; Grand Island, NY) and imaged with a Zeiss LSM 510 laser scanning confocal microscope (Carl Zeiss Microscopy; Thornwood, NY).

**Table 1.** Antibodies Used in This Study.

Antigen	Species/Clone Name	Dilution	Source/Catalog No. or Reference	Secondary, Source
Collagen α1α2α1(IV)	goat	20 μg/ml	SouthernBiotech; Birmingham, AL/1340-01	donkey anti-goat IgG Alexa Fluor 488, chicken anti-goat IgG Alexa Fluor 594, Invitrogen
Collagen α3α4α5(IV)	mouse/26-20	50 μg/ml	Borza, D.-B./Heidet et al. 2003	goat anti-mouse IgG1 Alexa Fluor 488, goat anti-mouse IgG1 Alexa Fluor 594, Invitrogen
Laminin α1	rat/8B3	50 μg/ml	Abrahamson, D.R./Abrahamson et al. 1989	chicken anti-rat IgG Alexa Fluor 488, donkey anti-rat IgG Alexa Fluor 594, Invitrogen
Laminin α5	rabbit	1:200 dilution	Miner, J.H./Miner et al. 1997	chicken anti-rabbit IgG Alexa Fluor 488, donkey anti-rabbit IgG Alexa Fluor 594, Invitrogen

### Electron Microscopy

For immunoperoxidase electron microscopy, mouse anti-collagen α3α4α5(IV) monoclonal 26-20 IgG (Heidet et al. 2003) was directly conjugated to activated horseradish peroxidase (HRP) using methods previously described (Nakane and Kawaoi 1974). IgG-HRP conjugates (0.2 ml at 1.4 mg/ml) were injected intraperitoneally into newborn mice. Twelve hours later, mice were anesthetized, and kidneys were fixed in situ by cortical injection of 1.6% paraformaldehyde and 3% glutaraldehyde (Karnovsky 1965) in 0.1 M sodium phosphate buffer, pH 7.4. Kidneys were removed, processed for peroxidase histochemistry with diaminobenzidine and hydrogen peroxide as before (Abrahamson and St. John 1992), post-fixed with osmium tetraoxide, and embedded in epoxy. Ultrathin sections were briefly stained with lead citrate and examined in a JEOL JEM-1400 transmission electron microscope (JEOL USA; Peabody, MA).

### qPCR

Kidneys obtained from 3-day-old (*N*=3) and 5-week-old (*N*=3) mice were promptly immersed in RNA*later* RNA stabilization reagent (Qiagen; Valencia, CA) and then stored at −80C. Total kidney RNA was collected using RNeasy (Qiagen) and incubated with primers designed to hybridize specifically with mouse Lama1, Lama5, Lamb1, Lamb2, Lamc1, Col4a1, Col4a2, Col4a3, Col4a4, Col4a5, and Col4a6 mRNAs (Table 2), and Quantitect SYBR Green RT-PCR reagents (Qiagen). Products were amplified and quantified in an iCycler (BioRad; Hercules, CA). Relative RNA abundance was calculated using the comparative Ct method (Livak and Schmittgen 2001).

**Table 2.** qPCR Primers Used in This Study.

Gene Symbol/Accession	Primer Sequence	Product Length (bp)
Lama1/NM_008480.2	FOR: 5’-CCGACAACCTCCTCTTCTACC-3’	60
	REV: 5’-TCTCCACTGCGAGAAAGTCA-3’	
Lama5/NM_001081171.2	FOR: 5’-ACCCAAGGACCCACCTGTAG-3’	169
	REV: 5’-TCATGTGTGCGTAGCCTCTC-3’	
Lamb1/NM_008482.1	FOR: 5’-GGCAAACTGCAAAGTCTCG-3’	61
	REV: 5’-CTGGAGGTGTTCCACAGGTC-3’	
Lamb2/NM_008483.3	FOR:5’-GTGTGGCTTGCATAGCCCT-3’	122
	REV: 5’-TCCGATGACTATTTGGGTTGTCT-3’	
Lamc1/NM_010683.2	FOR: 5’-TGCCGGAGTTTGTTAATGCC-3’	185
	REV: 5’-CTGGTTGTTGTAGTCGGTCAG-3’	
Col4a1/NM_009931.2	FOR: 5’-CTGGCACAAAAGGGACGAG-3’	238
	REV: 5’-ACGTGGCCGAGAATTTCACC-3’	
Col4a2/NM_009932.3	FOR: 5’-TGCTACCCGGAGAAAGGAG-3’	106
	REV: 5’-CTTTGCGGCCCTGTAGTCC-3’	
Col4a3/NM_007734.1	FOR: 5’-GGGACATGTAACTACTACTCAAACTCC-3’	91
	REV: 5’-CAGTTGATGGAATAGGTTTTCTGA-3’	
Col4a3 (alt)/NM_007734.1	FOR: 5’- AGTCCATGCACCGAGTGTC-3’	103
	REV: 5’- CAGGCACACCTCGTCCTC-3’	
Col4a4/NM_007735.1	FOR: 5’-CTGGCTTGAAGGGAGACCT-3’	69
	REV: 5’-CTCCTGCATCACCAGGAAGT-3’	
Col4a4 (alt)/NM_007735.1	FOR: 5’-TGGAGGAGTCTCTGGATTGG-3’	90
	REV: 5’-GCTGCCAGGTGACATCAGT-3’	
Col4a5/NM_007736.4	FOR: 5’-GGAGAACGGGGGTTTCCAG-3’	247
	REV: 5’-CTCCCTTGGTTCCATTGCATC-3’	
Col4a6/NM_053185.1	FOR: 5’-GACCATATGGATCAAAAGGAGATAAG-3’	77
	REV: 5’-GTGGCCCGGAATACCACT-3’	
Ppia/NM_008907	FOR: 5’-CAGACGCCACTGTCGCTTT-3’	132
	REV: 5’-TGTCTTTGGAACTTTGTCTGCAA-3’	

## Results

### Dual Immunolocalization of Laminin α1 and Laminin α5

**Figure 1.**
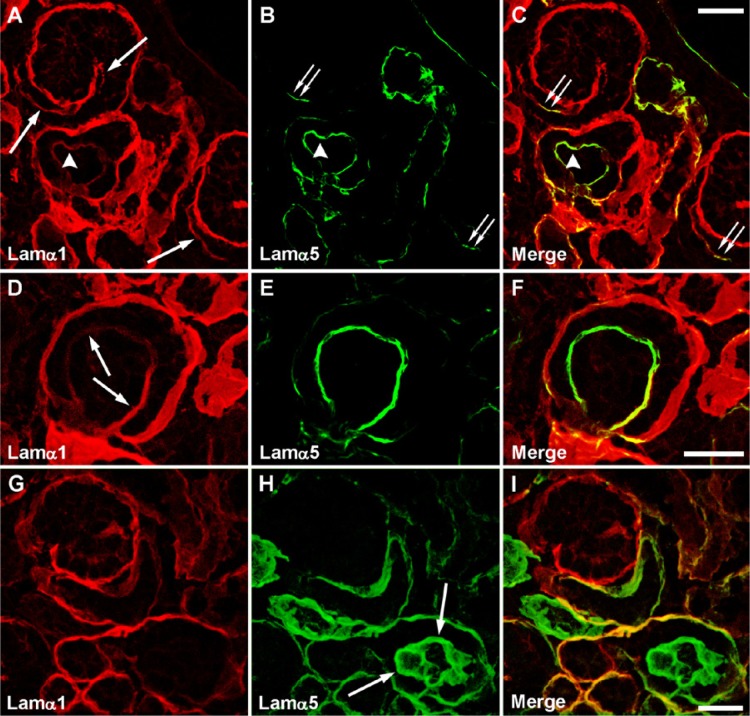
Dual label scanning confocal immunofluorescence microscopy of newborn mouse kidney sections showing the distribution of laminin α1 (Lamα1, red), laminin α5 (Lamα5, green), and both proteins (Merge), in developing glomerular basement membranes (GBMs). (A) Laminin α1 is present throughout the vascular cleft basement membrane (arrows) of comma- and S-shaped glomeruli. In contrast, only short stretches of these same basement membranes also contain laminin α5 (double arrows in B and C). GBMs of early capillary loop stage glomeruli contain both laminin α chains simultaneously (arrowheads in A–C), but the signal for laminin α1 appears to fade as glomerular development progresses (compare arrowed sites in D). (G–I) As additional capillary loops form, GBMs contain abundant laminin α5 (arrows) but laminin α1 is absent. Bars = 20 µm.

**Figure 2.**
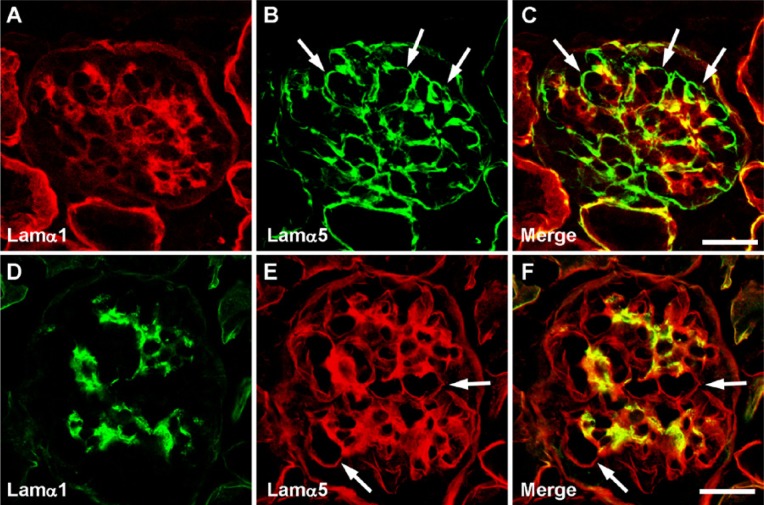
Maturing stage glomerulus from a newborn mouse (A–C) and fully mature glomerulus from an 8-week-old mouse (D–F), dually immunolabeled for laminin α1 and laminin α5, as indicated. In both cases, peripheral loop glomerular basement membranes contain only laminin α5 (arrows), whereas mesangial matrices contain laminin α1 and α5 (yellow in merged images C and F). Bars = 20 µm.

**Figure 3.**
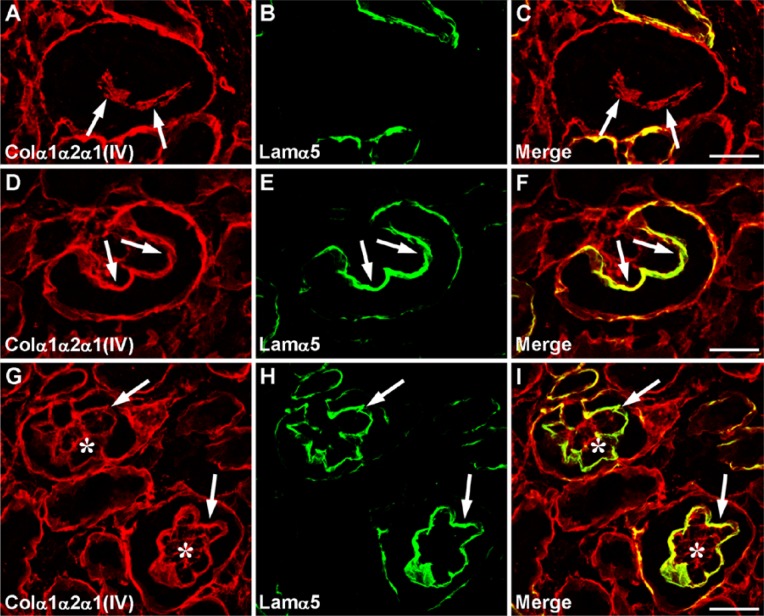
Newborn mouse kidney sections dually immunolabeled with anti-collagen α1α2α1(IV) [Colα1α2α1(IV), red] and anti-laminin α5 (green). (A–C) The earliest glomerular basement membranes (GBMs) of vascular clefts contain collagen α1α2α1(IV) (arrows) but no laminin α5 (compare with Figs. 1A–1C). (D–F) At the early capillary loop stage, there is overlapping distribution of collagen α1α2α1(IV) with laminin α5 (arrows). (G–I) As capillary loop stage glomeruli continue to progress, collagen α1α2α1(IV) codistributes with laminin α5 in GBMs (arrows). Collagen α1α2α1(IV) can also be seen in developing mesangial matrices (*). Bars = 20 µm.

**Figure 4.**
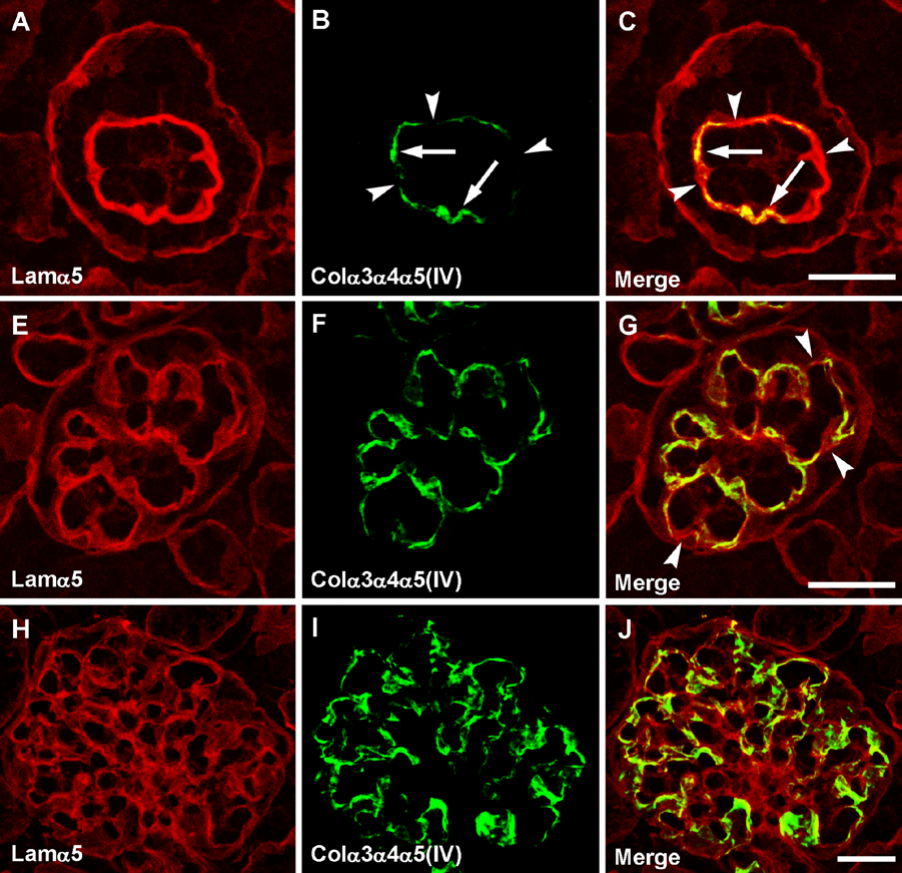
Laminin α5 is distributed in linear, continuous patterns in early and later stage capillary loop glomerular basement membranes (GBMs) (A–G), as well as throughout GBMs and mesangial matrices of maturing stage glomeruli (H–J). Collagen α3α4α5(IV) is first observed in early capillary loop GBMs in focal, discontinuous patterns (B–C, arrows) that are interrupted by GBM segments that are positive for laminin α5 (B–C, arrowheads). As more capillary loops are added to developing glomeruli, more collagen α3α4α5(IV) is deposited, but lengths of laminin α5 positive GBM lack collagen α3α4α5(IV) (G, arrowheads). GBMs of maturing stage glomeruli of newborn kidneys are still not fully labeled with anti-collagen α3α4α5(IV) (H–J). Bars = 20 µm.

When cryosections from newborn mouse kidney were doubly immunolabeled with anti-laminin α1 and anti-laminin α5 IgGs, basement membranes within the vascular clefts of comma- and early S-shaped figures, representing the earliest GBMs, contained predominantly laminin with an α1 chain (Figs. 1A–1C). In contrast, there were only short stretches of laminin α5 labeling found within these same basement membranes (Figs. 1A–1C). However, beginning in the early capillary loop stage, peripheral loop GBMs contained abundant laminin α5 labeling in linear patterns, and the presence of the laminin α1 chain began to fade (Figs. 1A–1F). As capillary loops were added to glomeruli, and in all later stages of glomerular development, GBMs were brightly labeled with anti-laminin α5 in strong linear patterns (Figs. 1–4), and GBMs became completely negative for the laminin α1 chain (Figs. 1G–1I and Fig. 2). Therefore, as seen earlier by immunoperoxidase staining of separate sections (St. John and Abrahamson 2001), these dual label immunofluorescence results from the same section demonstrate that the laminin α1-to-α5 switch occurred early in glomerular development. Additionally, the downregulation of laminin α1 and upregulation of laminin α5 were abrupt, and there were only short segments of developing GBM within the earliest nephric figures containing laminin α1 and α5 chains simultaneously. In maturing stage glomeruli of newborn mice, as well as in all glomeruli of 8-week-old mice, peripheral loop GBMs contained solely laminin α5 (Fig. 2). In mesangial matrices of maturing glomeruli in newborns, and in fully mature glomeruli of adults, however, there was partial signal overlap for both laminin α1 and α5 chains (Fig. 2).

### Dual Immunolocalization of Laminin α5 and Type IV Collagen

Knowing that laminin isoform transitioning occurred early in glomerular development, we next sought to compare this with type IV collagen expression. When sections were dually labeled with anti-collagen α1α2α1(IV) and anti-laminin α5, vascular cleft GBMs of the earliest nephric figures were positive for collagen α1α2α1(IV) and negative for laminin α5 (Figs. 3A–3C). When capillary loop stage glomeruli were examined, anti-collagen α1α2α1(IV) and anti-laminin α5 both labeled developing peripheral loop GBMs in strong linear patterns with considerable signal overlap (Figs. 3D–3I).

Whereas expression of collagen α1α2α1(IV) occurred within GBMs from the inception of glomerulogenesis, the expression of collagen α3α4α5(IV) was not observed until the capillary loop stage and progressed gradually thereafter as new glomerular capillary loops emerged. Sections dually labeled with anti-laminin α5 and anti-collagen α3α4α5(IV) showed linear, laminin α5-positive GBMs in developing capillary loops that contained discontinuous segments labeled with anti-collagen α3α4α5(IV) (Figs. 4A–4G). As glomeruli matured and acquired more capillary loops, their GBMs became increasingly positive for anti-collagen α3α4α5(IV) (Figs. 4H–4J). However, compared to the consistent, linear labeling patterns for laminin α5 throughout all capillary loop stage and maturing glomeruli GBMs, collagen α3α4α5(IV) appeared in discontinuous, fragmentary patterns, even in maturing stage glomeruli of newborn kidney (Figs. 4H–4J).

### Dual Immunolocalization of Collagen α1α2α1(IV) and Collagen α3α4α5(IV)

**Figure 5.**
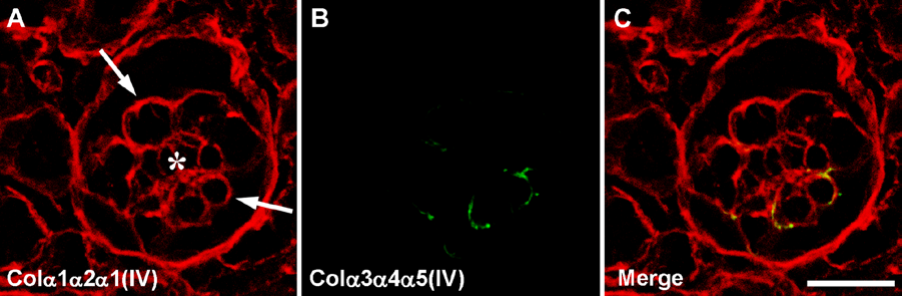
Localization of collagen α1α2α1(IV) and α3α4α5(IV) in a capillary loop stage glomerulus. Dually immunolabeled section shows collagen α1α2α1(IV) in loop glomerular basement membranes (A, arrows) and developing mesangial matrix (*); collagen α3α4α5(IV) is seen only in fragmentary, discontinuous patterns in a few loops (arrows, B, C). Bars = 20 µm.

**Figure 6.**
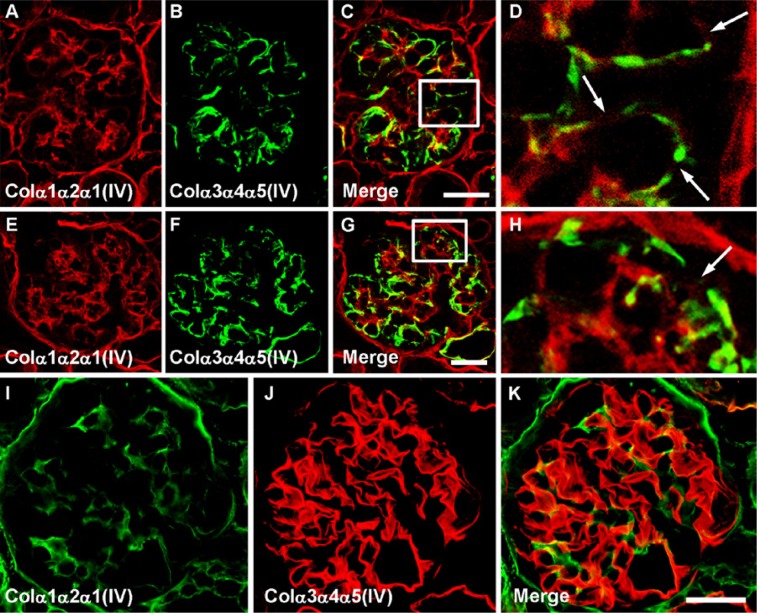
Localization of collagen α1α2α1(IV) and α3α4α5(IV) in maturing stage glomeruli of newborn (A–H) and 8-week-old mice (I–K). In maturing stage glomeruli of newborns, there is very little signal overlap in glomerular basement membranes (GBMs) or mesangial matrices for these two type IV collagen isoforms (C and G). Boxed areas in C and G are shown in D and H, respectively, where there are overt discontinuities or gaps in labeling for either isoform of type IV collagen in peripheral loop GBMs (arrows). In fully mature glomeruli of 8-week-old kidneys, anti-collagen α1α2α1(IV) immunolabels only mesangial matrices, whereas anti-collagen α3α4α5(IV) labels GBMs exclusively in a continuous, linear pattern. Unlike the case for laminin α1 and laminin α5, there is essentially no signal overlap for these different collagen IV networks in mesangial areas (compare with Figs. 2C and 2F). Bars = 20 µm.

To compare directly the distribution patterns of the two type IV collagen isoforms, newborn kidney that had been dually labeled with anti-collagen α3α4α5(IV) and anti-α3α4α5(IV) produced some unexpected findings. First, there was almost no signal overlap for these two different collagen IV isoforms either in capillary loop GBMs or in developing mesangial matrices of maturing glomeruli in newborns (Figs. 5 and 6), or in fully mature glomeruli of adult, 8-week-old mice (Figs. 6I–6K). Second, collagen α3α4α5(IV) was again seen only in discontinuous patterns in peripheral loop GBMs of developing glomeruli (Figs. 5 and 6A–6H). Third, when these GBMs were examined carefully, there were overt gaps in labeling where there was a complete absence of type IV collagen in the GBM altogether (Figs. 6A–6H). When fully mature glomeruli of 8-week-old mice were examined, however, capillary loop GBMs were now completely labeled with anti-collagen α3α4α5(IV) in continuous, linear patterns, whereas collagen α1α2α1(IV) was seen only in mesangial matrices (Figs. 6I–6K). When slides of developing or mature kidney were incubated with mixtures of secondary antibodies alone as controls, all of the sections were consistently negative.

### Immunoelectron Microscopic Localization of Collagen α3α4α5(IV)

**Figure 7.**
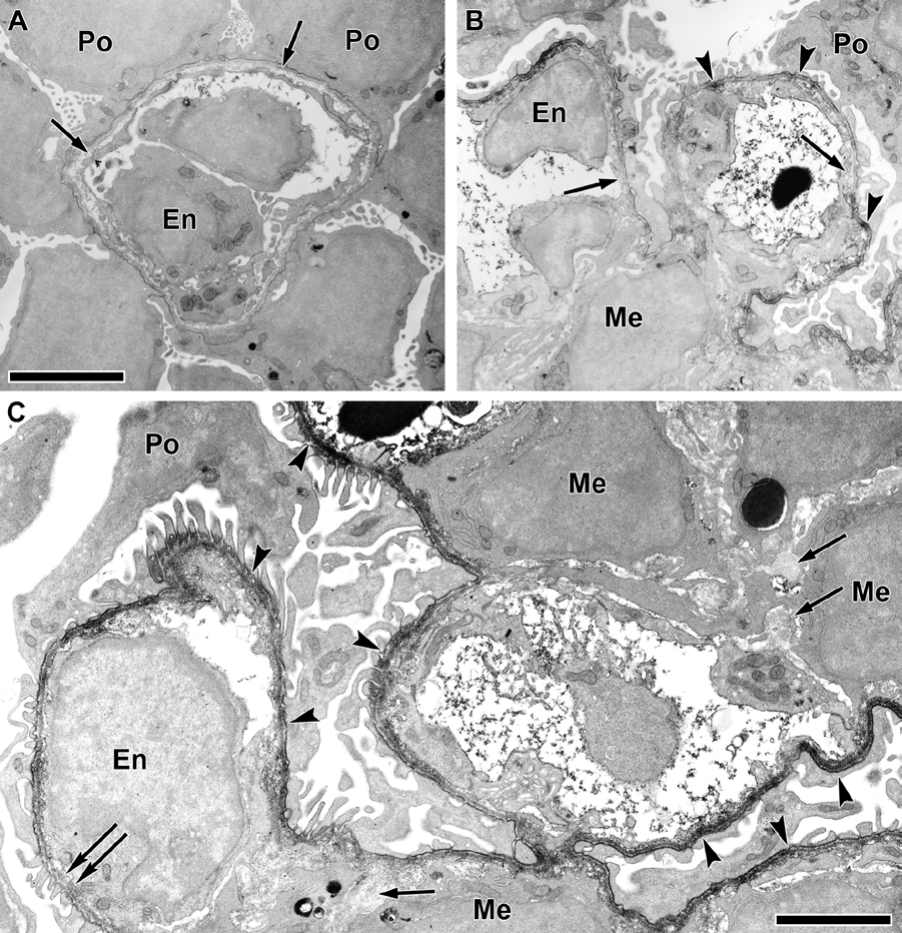
Immunoelectron microscopic localization of collagen α3α4α5(IV) in newborn mouse kidney. (A) Glomerular basement membranes (GBMs) of early capillary loop stage glomeruli are unlabeled (arrows). (B) In later stage capillary loop GBMs, discontinuous, uneven deposits of peroxidase reaction product (arrowheads) can be seen in GBMs that also contain unlabeled segments (arrows). (C) Maturing stage glomeruli contain GBMs that appear more uniformly labeled (arrowheads), although there are areas that remain spotty (double arrow). Note that reaction product is absent from mesangial matrices (arrows). En, endothelium; Po, podocyte; Me, mesangium. Bars A and B = 10 µm; bar C = 3 µm.

To extend these findings further, we injected anti-collagen α3α4α5(IV) IgG-HRP conjugates into newborn mice, and kidneys were fixed and processed for peroxidase histochemistry and electron microscopy. As shown in Fig. 7A, there was a complete absence of HRP reaction product in most GBMs of early capillary loop stage glomeruli, which was consistent with our immunofluorescence results showing no immunoreactivity for anti-collagen α3α4α5(IV) in the earliest GBMs. In later capillary loop stages, however, marked by the presence of forming podocyte foot processes, peroxidase reaction product could be observed within developing GBM in patchy, discontinuous patterns (Fig. 7B). In contrast, maturing stage glomeruli contained capillary loop GBMs with linear, almost homogeneous reaction product deposits, although there were lengths where little labeling was present (Fig. 7C). Mesangial matrices, however, were consistently negative for anti-collagen α3α4α5(IV) IgG-HRP (Fig. 7C), which was also consistent with our confocal results showing an absence of this collagen isoform in the mesangium (Fig. 6). As shown previously in control experiments, there is no glomerular binding in mice injected with normal IgG-HRP conjugates (Abrahamson et al. 1989; Abrahamson and St. John 1992). Additionally, previous studies with this particular anti-collagen α3α4α5(IV) IgG-HRP conjugate have shown that it does not bind to GBMs of Alport mouse kidneys, which lack the collagen α3α4α5(IV) network, but does bind to wild-type mouse glomeruli, further documenting its specificity (Abrahamson et al. 2009).

### Developmental Changes in Kidney Laminin and Collagen IV mRNAs

**Figure 8.**
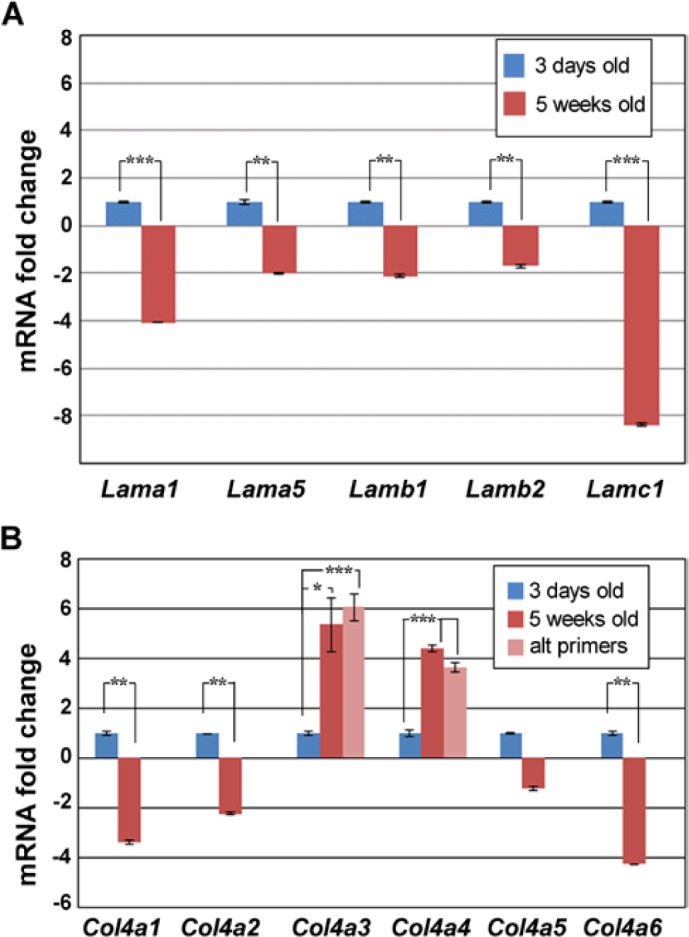
Histograms showing relative expression levels for mRNAs encoding laminin (A) and type IV collagen (B) chains in kidneys from 3-day-old (blue) and 5-week-old (red) mice. As seen in A, compared with newborns, all of the laminin chain mRNAs are significantly downregulated in mature mice. In contrast, mRNAs encoding the α3 and α4 chains of type IV collagen are significantly more abundant in mature kidneys, whereas those encoding collagen α1(IV), α2(IV), and α6(IV) chains are downregulated (B). *p<0.05, **p<0.005, and ***p<0.0005. Light red bars = alternate (alt) primers for COL4A3 and COL4A4 (Table 2), confirming increase for these mRNAs at 5 weeks of age.

To evaluate comparative expression levels for laminin and collagen IV in developing and mature kidney, we undertook a quantitative PCR analysis of mRNAs isolated from 3-day-old and 5-week-old mouse kidneys (*N*=3 at each age). Compared with levels in 3-day-old kidneys, mRNAs encoding Lama1, Lama5, Lamb1, and Lamc1 gene expression were all significantly lower in 5-week-old kidneys, with deceases ranging from ~3-fold to >9-fold (Fig. 8A). Messenger RNAs encoding Col4a1, Col4a2, Col4a3, Col4a4, Col4a5, and Col4a6 were also evaluated. Messages for Col4a1, Col4a2, and Col4a6 all declined significantly in 5-week-old kidneys (Fig. 8B). By contrast, mRNAs for COL4A3 and COL4A4 were significantly upregulated in 5-week-old kidneys compared to those from 3-day-old mice (~6-fold, Fig. 8B), and this was confirmed using two different sets of primers for these gene products (Table 2).

## Discussion

The main findings from this immunolocalization study of developing glomeruli are that the laminin and collagen IV isoform substitutions occurring during GBM assembly were spatially and temporally distinct. Laminins containing the α1 chain were abruptly replaced with those containing laminin α5 at early capillary loop stages and all new GBMs that formed subsequently contained laminin α5 in continuous, linear patterns. In contrast, collagen IV substitution began later, in more advanced glomeruli, where synthesis of collagen α1α2α1(IV) was slowly replaced by collagen α3α4α5(IV), which appeared in discontinuous, non-linear patterns in GBMs. The two collagen IV isoforms generally did not occur together within the same GBM segments, however, and there were lengths of continuously labeled, laminin α5-positive GBM that were unlabeled by either anti-collagen α1α2α1(IV) or anti-collagen α3α4α5(IV). Additionally, qRT-PCR showed that whereas the relative abundance of mRNAs encoding laminin α1, α5, β1, β2, and γ1, and collagen α1(IV), α2(IV), and α6(IV) chains were markedly decreased in mature kidney, those encoding the collagen α3(IV) and collagen α4(IV) chains were unexpectedly and significantly increased.

Immunofluorescence microscopy of developing kidney using isoform-specific antibodies has previously shown that laminin α1, β1, and collagen α1α2α1(IV) are present in GBMs of the earliest glomeruli, and laminin α5, β2, and collagen α3α4α5(IV) are in GBMs of mature glomeruli (Miner and Sanes 1994; Miner et al. 1997; Sorokin et al. 1997). However, our findings presented here represent the first report that we are aware of to simultaneously immunolocalize the different laminin and collagen IV isoforms in developing GBMs. Our results clearly demonstrate that the specific isoform switches for laminin and collagen IV occurred on different developmental timetables. Downregulation of laminin α1 and upregulation of laminin α5 deposition occurred early in glomerular development, whereas downregulation of collagen α1α2α1(IV) and upregulation of α3α4α5(IV) deposition occurred later. Additionally, the patterns for laminin and collagen IV transitioning were strikingly different. Once the laminin α1–α5 switch occurred in early capillary loop stage glomeruli, all of the GBM assembled thereafter contained laminin α5 in linear patterns. In contrast, the deposition of collagen α3α4α5(IV) into GBMs of newborn kidney took place over a gradual period and in non-linear patterns, leaving segments of laminin α5-positive GBM in maturing glomeruli that lacked collagen IV labeling entirely. In fully mature glomeruli of 8-week-old mice, however, all of the GBMs were immunolabeled with anti-collagen α3α4α5(IV) in linear, continuous patterns.

Previous work has shown that ultrastructurally normal basement membranes can form in Col4a1/2 knockout embryos, which survive until mid-gestation in the complete absence of collagen IV (Poschl et al. 2004). The authors of that study speculated that the presence of laminin and other basement membrane proteins in early embryos allows for blastocyst formation, implantation, and initial organogenic events. Beginning in mid-gestation, however, basement membranes in Col4a1/2-null embryos degrade, probably because they are inadequately stabilized due to the absence of a collagen α1α2α1(IV) network (Poschl et al. 2004). Similarly, in our results presented here, the laminin α5-positive peripheral loop GBMs lacking collagen IV are presumably able to function adequately until they are later modified by the addition of collagen α3α4α5(IV). On the other hand, if collagen α3α4α5(IV) is never installed in the GBM, as is the case in Alport syndrome, the glomerulus eventually loses its barrier properties and the GBM disintegrates (Hudson et al. 2003).

We previously provided evidence that laminin α1 and β1, and subsequently laminin α5 and β2, are all synthesized and secreted by both endothelial cells and podocytes in developing glomeruli (St. John and Abrahamson 2001). Additionally, we showed that when fetal kidneys from laminin α5 knockout mice are grafted into kidney cortices of newborn wild-type hosts, host-derived endothelial cells migrate into grafts and establish hybrid glomeruli containing wild-type endothelial cells and Lama5-null podocytes (Abrahamson et al. 2007). Glomerular basement membranes within these hybrid glomeruli are abnormal, however, and contain laminin α5 only on the inner surfaces facing the wild-type endothelium and retain laminin α1 on their outer surfaces beneath the laminin α5 mutant podocytes. At the ultrastructural level, these hybrid GBMs are poorly condensed and the podocytes fail to form foot processes (Abrahamson et al. 2007). At the time, we speculated that the laminin α1–α5 switch was important for inducing podocyte differentiation and foot process formation (Abrahamson et al. 2007). Because glomerular collagen α3α4α5(IV) is synthesized only by podocytes (Abrahamson et al. 2009), perhaps the laminin α1–α5 switch occurs first to induce podocytes to differentiate further, which then activates the collagen α1α2α1(IV)–α3α4α5(IV) transition. If true, this would help explain the temporal delay in collagen α3α4α5(IV) deposition. What is not at all clear, however, is how the collagen α3α4α5(IV) network is integrated into the laminin network to constitute the mature GBM, and much more work needs to be carried out at the cellular and biochemical levels in order to understand this process fully.

We emphasize that the comparative expression differences that we observed for laminins and type IV collagen between 3-day-old and 5-week-old whole kidney mRNAs may not accurately reflect changes occurring only in glomeruli, which probably represent less than 5% of the total kidney tissue mass. Although we now routinely isolate glomeruli from mice as young as 18 days old using the magnetic bead perfusion technique (Takemoto et al. 2002), we have not successfully applied this approach to enrich for glomeruli from newborn mice, which is why we used whole kidney RNA samples for the comparative expression experiments described here. Nevertheless, the significant reductions in mRNAs encoding the laminins, and COL4A1 and COL4A2, were not surprising, as glomeruli and tubules in 5-week-old mouse kidneys have reached their fully mature dimensions, and GBM and TBM basement membrane assembly should be complete. Moreover, basement membranes in mature kidneys are also highly stable structures, and metabolic labeling studies show that rat GBM collagen turnover has a half-life estimated to be greater than 100 days (Price and Spiro 1977), which would largely obviate the need for laminin and collagen IV gene transcription. On the other hand, that Col4a3 and Col4a4 mRNAS were significantly upregulated in 5-week-old kidneys compared with 3-day-old kidneys was an unexpected finding that we verified using an independent set of qPCR primers. The COL4A3 and COL4A4 genes are located on mouse chromosome 1 and are organized in a head-to-head conformation (Sado et al. 1998; Lu et al. 1999). The tandem expression of both genes was therefore likely due to their sharing a common promoter, although the exact regulatory mechanisms for any of the collagen IV genes, as well as those for the laminins, are extremely poorly understood.

In summary, we have described the developmental timing for the GBM laminin and collagen IV isoform substitutions, and these processes are temporally and spatially distinct. Taking all of the results together, we conclude that the biosynthesis of laminin and collagen IV is probably regulated by very different molecular genetic controls. Similarly, the installation of the various laminin and collagen IV protein isoforms into the GBM and their removal are most likely also mediated by different mechanisms. Nevertheless, these processes surely must be coordinated to ensure that the glomerular filtration barrier is assembled properly. What we need to learn next are how the various laminin and collagen IV genes are sequentially silenced and activated and how the early protein isoforms are replaced in maturing GBM. This information will be important for understanding and potentially treating fibrotic diseases such as glomerulosclerosis, where there is overproduction and accumulation of basement membrane proteins.
